# Non-convulsive seizures in the encephalopathic critically ill cancer patient does not necessarily portend a poor prognosis

**DOI:** 10.1186/s40560-019-0414-0

**Published:** 2019-12-16

**Authors:** Cristina Gutierrez, Merry Chen, Lei Feng, Sudhakar Tummala

**Affiliations:** 1 Critical Care Department, Division of Anesthesia and Critical Care, The University of Texas MD Anderson Cancer Center, 1515 Holcombe Blvd, unit 112 Room B7.4320, Houston, TX 770130 USA; 2 Department of Neuro-Oncology, Division of Cancer Medicine, The University of Texas MD Anderson Cancer Center, Houston, TX USA; 3 Department of Biostatistics, Division of Quantitative Sciences, The University of Texas MD Anderson Cancer Center, Houston, TX USA; 4 Department of Neuro-Oncology, Division of Cancer Medicine, The University of Texas MD Anderson Cancer Center, Houston, TX USA

**Keywords:** Encephalopathy, Cancer, Critically ill, Non-convulsive seizures, Status epilepticus, Mortality

## Abstract

**Background:**

Non-convulsive status epilepticus (NCSE) is present in 10–30% of ICU patients with altered mental status (AMS) and is associated to poor outcomes. To our knowledge, there is no data describing the prevalence and outcomes of critically ill cancer patients with AMS associated to non-convulsive seizures (NCS) or NCSE. We aim to describe the outcomes and risk factors of critically ill cancer patients with encephalopathy associated with non-convulsive seizures (NCS).

**Methods:**

This is a 3-year prospective observational study in a mixed oncological ICU at MD Anderson Cancer Center. Data of ICU patients with moderate to severe encephalopathy (Glasgow Coma Score < 13) that underwent EEG monitoring to rule out NCS were collected. Multivariate logistic regression was performed to identify risk factors and outcomes.

**Results:**

Of the 317 patients with encephalopathy who underwent EEG monitoring, 14.5% had NCS. Known risk factors such as sepsis, CNS infection, antibiotics, and cardiac arrest were not associated with increased risk of NCS. Patients with NCS were more likely to have received recent chemotherapy (41.3% vs 21.4%; *p* = 0.0036), have a CNS disease (39% vs 24.4%; *p* = 0.035), and abnormal brain imaging (60.9% vs 44.6%; *p* = 0.041). Patients with lower SOFA scores, normal renal function, and absence of shock were likely to have NCS as the cause of their encephalopathy (*p* < 0.03). After multivariate analysis, only abnormal brain imaging and absence of renal failure were associated with NCS. Mortality was significantly lower in patients with non-convulsive seizures when compared to those without seizures (45.7% vs 64%; *p* = 0.022); however, there was no significant association of seizures and mortality on a multivariable logistic regression analysis.

**Conclusions:**

NCS in critically ill cancer patients is associated with abnormalities on brain imaging and lower prevalence of organ failure. Diagnosis and treatment of NCS should be a priority in encephalopathic cancer patients, as they can have lower mortality than non-seizing patients. Opposite to other populations, NCS should not be considered a poor prognostic factor in critically ill encephalopathic cancer patients as they reflect a reversible cause for altered mentation.

## Background

Non-convulsive seizures (NCS) and non-convulsive status epilepticus (NCSE) can be a cause of coma and altered mentation in 18 to 45% of patients admitted to the Intensive Care Unit (ICU) [1–4]. Electroencephalogram (EEG) monitoring is frequently used as part of the diagnostic work up of encephalopathic critically ill patients [5]. Patients diagnosed with seizures in the ICU usually have worse outcomes, including increased length of stay (LOS) and mortality, and increased ICU costs [5–8]. Moreover, they can have long-term medical consequences as more than 60% of patients diagnosed with NCS and NCSE have recurrent seizures after being discharged from the hospital [9]. If the status epilepticus is not treated promptly, the response to therapy can decrease from 80 to 30%; therefore, early diagnosis and aggressive treatment is the cornerstone of improving outcomes in these patients [10, 11].

In the cancer population, 13% of patients experience seizures at some point during the course of their disease, and the prevalence of NCSE in the cancer population is 6–8% [12, 13]. Seizures are the most common neurological complication found in oncological ICUs, and neurological complications in cancer patients carry significant mortality [13, 14]. The etiologies of seizures in oncological patients are similar to those in the general population as follows: poor-compliance with medications, alcohol intoxication or withdrawal, infections, stroke, central nervous system tumors (primary or metastatic), trauma and anoxic encephalopathy [12, 13]. The causes and risk factors specific to the oncological population, such as the use of specific chemotherapeutic regimens, should also be considered when assessing these patients.

Due to the high morbidity and mortality associated with NCS and NCSE, early recognition in the encephalopathic patient is extremely important. Studies that help to identify patients at risk can lead to early diagnosis and possibly improve outcomes. To our knowledge, there are no published data describing characteristics and outcomes of NCS and NCSE in ICU cancer patients with moderate to severe encephalopathy.

## Materials and methods

**Fig. 1:**
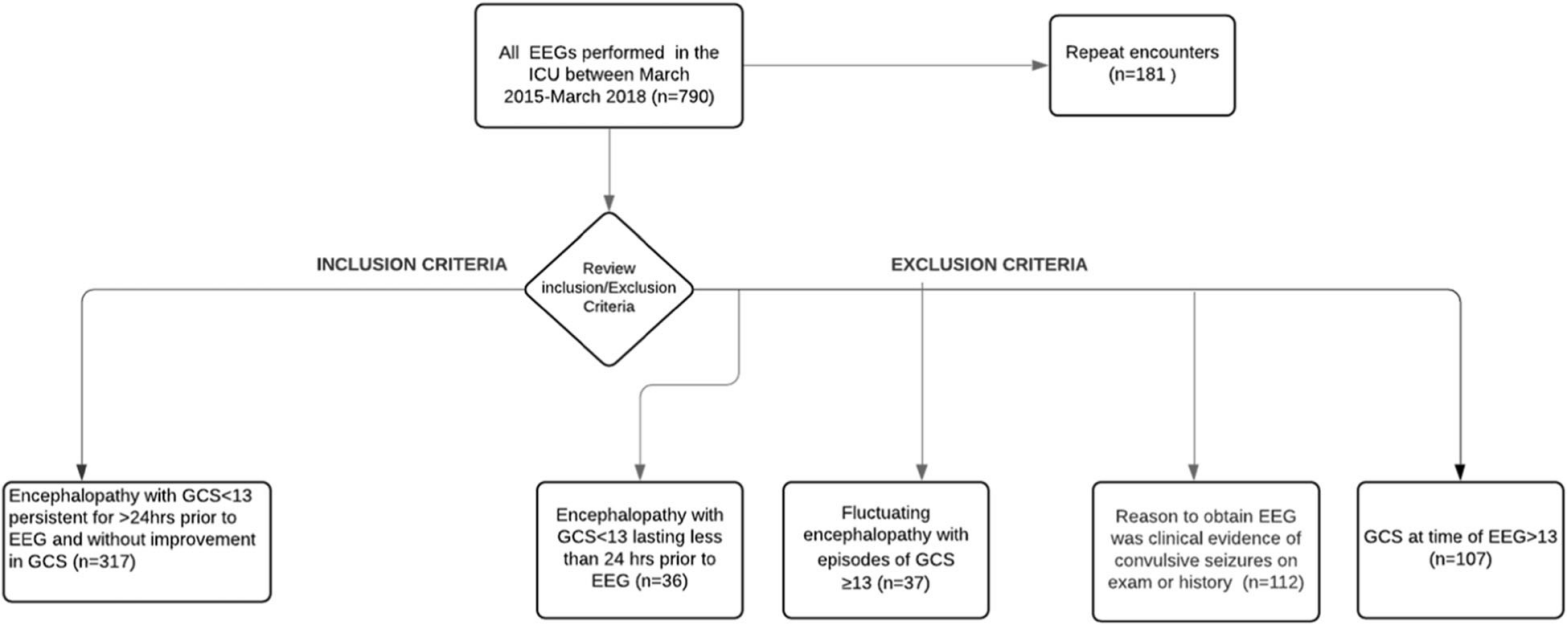
Inclusion and exclusion criteria

We conducted a prospective observational study during a 3-year period between March 2015 to March 2018, in our mixed surgical and medical ICU. The Institutional Review Board at MD Anderson Cancer Center (PA15-0304) approved the study. We included data of all patients who underwent EEG monitoring to diagnose NCS as the cause of encephalopathy and altered mental status (AMS). All patients included in our study had moderate to severe encephalopathy and depressed level of consciousness, measured as a Glasgow Coma Score (GCS) less than 13. Additionally, GCS < 13 had to be persistent for more than 24 h, non-fluctuating and not improving. Patients younger than 18 years of age, without cancer, with a GCS ≥ 13, and who underwent EEG monitoring because history and physical exam suggested convulsive seizures, were excluded from this study (Fig. 1). In patients who underwent multiple EEGs during the same or different ICU admissions, only the initial encounter was included in the analysis.

Demographic, clinical, and seizure-related data were collected in Redcap [15]. EEG monitoring in our institution begins with an initial 20–40 min EEG read by the epileptologist (authors ST or MC—both board certified in epilepsy). If deemed necessary, the patient remains on EEG for long-term monitoring either because of a pattern for NCSE and or the patient is not improving. Non-convulsive seizures and non-convulsive status epilepticus are identified in our institution by published criteria [5, 16]. Other patterns such as generalized and focal slowing, cortical suppression, burst suppression, hemisphere disturbance and its degree, periodic discharges of triphasic or biphasic morphology, spike and wave, sharp and wave, polyspike and wave, and electrographic seizures were documented. The use of anti-epileptic drugs (AEDs) and their response was documented. Risk factors for seizures reported in the literature such as sepsis, antibiotics, chimeric antigen receptor (CAR)T-cell therapy, intracranial tumors, history of seizures, central nervous system (CNS) infections, anoxic brain injury, hemorrhagic and ischemic strokes, posterior reversible encephalopathy syndrome (PRES), and electrolyte disturbances were documented [5, 12, 13, 17–19]. Medication use associated with seizures specific to the oncological population including methotrexate, mycophenolic acid, tacrolimus, cisplatin, busulphan, cytarabine, thiotepa, etoposide, chlorambucil, 5-fluorouracil, cyclosporine, carmustine, and paclitaxel were also documented [13]. The degree of organ failure was measured by the Sequential Organ Failure Assessment (SOFA) score, and independently by the use of vasopressors, mechanical ventilation and presence of renal failure. Outcomes measured included prevalence of NCS, response to AEDs, improvement of GCS prior to discharge (defined as GCS back to baseline or ≥ 13), length of stay (LOS), and mortality.

### Statistical analysis

Summary statistics including mean, standard deviation, median, and range for continuous variables, frequency counts, and percentages for categorical variables are provided. Fisher’s exact test or chi-square test was used to evaluate the association between two categorical variables. Wilcoxon rank sum test was used to evaluate the difference in a continuous variable between patient groups. Multivariable logistic regression models were fitted to evaluate the effects of important covariates on the incidence of non-convulsive seizures and mortality. The full model included the covariates which had a *p* value < 0.2 from the univariable analysis, and a backward selection method was used to find the final model. Statistical software SAS 9.4 (SAS, Cary, NC) was used for analyses.

## Results

### General characteristics of the critically ill encephalopathic population

During the 3-year period, a total of 790 patients underwent EEG monitoring in our ICU and 324 patients met inclusion criteria for our study. None of the included patients had convulsive seizures. Seven patient records had multiple ICU admissions with EEG monitoring for which only the initial encounter was included, leaving 317 (40.1%) unique patient encounters for analysis. Patients were predominantly admitted to the medical ICU (89.3%), male (59.3%), had a comorbidity index ≥ 5 (68.5%), a hematologic malignancy (61.8%), and 31.6% had received a stem cell transplant (Table 1). The majority of patients had metastatic disease (72.7%), but only 26.5% of patients had central nervous system involvement such as primary or metastatic disease, leptomeningeal disease, or positive cytology for malignancy in cerebrospinal fluid. Abnormal findings on brain CT or MRI included intracranial hemorrhage (10.4%), ischemic stroke (11.7%), intracranial masses (18.6%), leptomeningeal disease (5.9%), and subdural hematoma (5.1%). The most common diagnoses on ICU admission were altered mental status (30.9%), respiratory failure (25.9%), and severe sepsis and septic shock (18.0%) (Table 1). At the time of ICU admission, only 37.5% of patients had a SOFA score ≥ 10, but at the time of EEG monitoring, more than half of the patients had a SOFA score ≥ 10 (50.8%). Multi-organ failure during ICU stay was common; mean SOFA score on admission was 8.5 ± 3.7 (median 8; 0–19) on admission, 79.5% of patients required mechanical ventilation, 52.4% had acute renal failure, and 56.5% had shock (56.5%) (Table 1). Almost half of patients required sedation prior to EEG monitoring (49.8%); 35.3% midazolam, 18.6% dexmetomidine, and 13.3% propofol. The average duration of sedation was 4.1 ± 4.3 days.

**Table 1:** Characteristics and outcomes of encephalopathic patients and those with and without non-convulsive seizures

Variable	All patients	Seizures*	No seizures*	*p* value
	(*n* = 317)	(*n* = 46)	(*n* = 271)	
General characteristics				
Age	60.2 ± 14.6, 62 (18–89)	59.3 ± 14.6, 62 (18–84)	60.4 ± 14.7, 62 (18–89)	0.775
Gender (male)	59.3%	43.5%	62.0%	*0.018*
Type of malignancy				0.609
Hematological	61.8%	65.3%	61.3%	
Solid tumor	38.2%	34.7%	38.7%	
Metastatic disease	72.7%	56.3%	75.2%	0.112
Active disease (no remission)	86.8%	86.9%	86.7%	0.965
Stem cell transplant (yes)	31.6%	26.1%	18.5%	0.284
Co-morbidity index ≥ 5	68.5%	63.0%	69.4%	0.393
Chemotherapy within 10 days	24.3%	41.3%	21.4%	*0.0036*
CNS malignancy involvement**	26.5%	39.0%	24.4%	*0.0358*
Medical history of seizure disorder	10.4%	13.0%	9.9%	0.527
Admission diagnosis				
Altered mental status	30.9%	60.9%	25.8%	*< 0.0001*
Respiratory failure	25.9%	4.3%	29.5%	
Severe sepsis/shock	18.0%	10.9%	19.2%	
Cardiac arrest	11.7%	10.9%	11.8%	
Stroke (hemorrhagic or ischemic)	2.8%	6.5%	2.2%	
Other	10.7%	6.5%	11.5%	
Variables during ICU stay				
Days from admission to EEG***	5.5 ± 7.5, 3 (1–69)	3.9 ± 5.3, 2 (1–30)	5.7 ± 7.8, 3 (1–69)	0.141
SOFA on admission^#^	8.5 ± 3.7, 8 (0–19)	7.7 ± 3.1, 7 (1–15)	8.6 ± 3.8, 9 (1–19)	0.170
SOFA at time of EEG^#^	9.9 ± 4.0, 10 (3–22)	8.5 ± 3.1, 8 (3–15)	10.2 ± 4.1, 10 (3–22)	*0.0129*
GCS at time of EEG (median)^	7 (3–12)	7 (3–12)	7 (3–12)	0.720
Vasopressors (yes)	56.5%	36.9%	59.8%	*0.0039*
Acute renal failure (yes)	52.4%	30.4%	56.1%	*0.0013*
Mechanical ventilation (yes)	79.5%	69.6%	81.2%	0.071
Use of sedatives (yes)	49.8%	36.9%	52.0%	0.059
Cardiac arrest during ICU stay	13.2%	10.9%	13.7%	0.814
Abnormal brain imaging	47.0%	60.9%	44.6%	*0.0416*
Meningitis/encephalitis	4.1%	8.7%	3.3%	0.103
Medications given in the ICU ^^	31.9%	39.1%	30.6%	0.253
Abnormal electrolytes	4.4%	4.3%	4.4%	1
PRES^+^	1.3%	2.2%	1.1%	0.467
CNS malignancy involvement	26.5%	39.0%	24.4%	*0.0358*
CAR T cell therapy^++^	4.7%	15.2%	2.9%	*0.0003*
Outcomes				
Improvement of GCS prior to discharge	44.2%	52.2%	42.8%	*0.237*
ICU Length of stay	13.8 ± 13.8	12.5 ± 11.5	14.1 ± 14.2	*0.655*
Hospital Length of stay	22.3 ± 21.2	21.3 ± 18.4	22.5 ± 21.7	*0.857*
Overall mortality	60.9%	45.7%	63.5%	*0.022*

*Non-convulsive seizures

**Central nervous system

***Electroencephalogram

^#^Sequential Organ Failure Assessment

^Glasgow Coma Score

^^ Medications known to cause seizures

^+^ Posterior reversible encephalopathy syndrome

^++^ Chimeric Antigen Receptor

Patients were admitted to the ICU for 5.5 ± 7.5 days before EEG monitoring and median GCS at the time was 7. Common findings on EEG were slowing (61.5%), periodic waves of triphasic and biphasic morphology (13.6%), and epileptiform sharp waves (14.5%). Twenty percent of EEGs had other findings such as diffuse hemisphere disturbance of mild to moderate degree, severe cortical suppression, and burst suppression patterns. Forty-six patients (14.5%) had intermittent NCSs on EEG; of these, 65.2% were in non-convulsive status epilepticus (*n* = 30). Benzodiazepines, levetiracetam, phenytoin, phosphenytoin, and phenobarbital were the most commonly used AEDs. Other AEDs included lacosamide, lamotrigine, and valproic acid. Eighty-seven percent of patients responded to the AEDs and 14 (46.7%) of the 30 patients who were in NCSE were considered refractory to more than two AEDs and required burst suppression.

### Risk factors for non-convulsive seizures

We further analyzed the data to discern risk factors associated to NCS in encephalopathic critically ill oncological patients. While patients with NCS were more likely to be female, other factors such as age, comorbidity index, type of malignancy, metastatic disease, and stem cell transplant status were not associated to NCS prevalence (*p* > 0.1) (Table 1). Patients with NCS were more likely to have received chemotherapy within 10 days of EEG monitoring (41.3% vs 21.4%; *p* = 0.0036); the prevalence of severe neutropenia was similar in patients with and without seizures (30.4% vs 23.6%; *p* = 0.32) (Table 1).

Patients with NCS were more likely to have been admitted to the ICU with a diagnosis of altered mental status, while those without seizures with respiratory failure and septic shock (*p* < 0.0001) (Table 1). Patients with NCS were less likely to require vasopressors (37.0 vs 59.8%; *p* = 0.039) and to have renal failure (30.4% vs 56.1%; *p* = 0.0013), and their SOFA at the time of EEG monitoring was significantly lower (8.5 ± 3.1 vs 10.2 ± 4.1; *p* = 0.0129) (Table 1). SOFA score on admission, GCS at the time of EEG, need for mechanical ventilation, the use of sedation, and its duration were similar in patients with and without NCS (Table 1). All patients with NCS had at least one risk factor described in the literature to cause seizures (100% vs 84.5%; *p* = 0.0015). Specific risk factors such as cardiac arrest, sepsis, history of seizures, meningitis/encephalitis, or PRES were not associated to an increased prevalence of NCS (Table 1). The use of specific medications, including specific seizure inducing chemotherapeutic agents and immunosuppressants, was not associated to an increased risk of NCS in our patient population (39.1% vs 30.6%; *p* = 0.25) (Table 1). Abnormal findings on brain CT or MRI (60.9% vs 44.6%; *p* = 0.0416), CNS malignancy involvement (39% vs 24.4%; *p* = 0.036) and undergoing recent CAR T cell therapy (15.2% vs 3.0%; *p* = 0.0003) were more prevalent in patients with NCS (Table 1). Subgroup analysis did not show any correlation between specific findings on brain CT or MRI and seizures (data not shown). After multivariate analysis, abnormalities on brain imaging, absence of renal failure, and recent chemotherapy were independently associated with an increased risk of non-convulsive seizures (Table 2). Concerning recent chemotherapy, since CAR T cell patients undergo chemotherapy as part of their lympho-depleting protocol prior to cell infusion, we excluded this patient population to evaluate if recent chemotherapy continued to be an independent risk factor for NCS. For the patients who did not receive CAR T cell therapy, chemotherapy was no longer associated with an increased risk of NCS (OR 2.03 for 95%CI = 0.94–4.42; *p* = 0.074).

**Table 2:** Multiple regression model of risk factors for non-convulsive seizures

Variables	Odds ratio	95% CI	*p* value
Female	1.82	0.94–3.54	0.078
CNS malignancy involvement	1.91	0.93–3.93	0.080
Use of sedatives	1.63	0.82–3.25	0.164
Chemotherapy within 10 days	2.95	1.47–5.94	*0.002*
Acute renal failure	2.23	1.08–4.61	*0.030*
Abnormal brain imaging	0.21	0.07–0.66	*0.007*

### Outcomes of encephalopathic critically ill patients and those with non-convulsive seizures

Overall, only 44.2% of all encephalopathic patients who underwent EEG monitoring had improvement of their GCS prior to hospital discharge. Moreover, recovery of GCS was similar between patients with and without NCS (52.2% vs 42.8%; *p* = 0.237) (Table 1). Improvement of GCS prior to discharge was associated with lower mortality (80.6% vs 20.7%; *p* < 0.0001). Encephalopathic cancer patients undergoing EEG monitoring in our ICU had prolonged LOS (ICU LOS: 13.8 ± 13.8 days and hospital LOS: 22.3 ± 21.2 days) and 60.9% mortality (Table 1). Factors associated to increased mortality in this patient population included the presence of metastatic disease with CNS involvement, being admitted to the ICU medical service and recent cardiac arrest (Table 3). Markers of multi-organ failure such as the need for mechanical ventilation and vasopressors, acute renal failure, lower GCS, and higher SOFA scores on admission were associated with increased mortality (*p* < 0.0001) (Table 3). After multivariable analysis, variables of organ failure such as vasopressors and renal failure were independently associated with mortality (Table 4). Surprisingly, mortality was lower in patients with NCS when compared to patients without seizures (45.7% vs 63.5%; *p* = 0.022); however, there was no significant association of seizures and mortality on multivariable logistic regression analysis (Table 1, Table 4). ICU and hospital LOS was similar in patients with and without NCS (Table 1).

**Table 3:** Mortality of all encephalopathic critically ill oncological patients

Variable	All patients	Survived	Dead	*p* value
	(*n* = 317)	(*n* = 193)	(*n* = 124)	
Age	60.2 ± 14.6, 62 (18–89)	58.9 ± 15.5, 62 (18–87)	61.1 ± 14, 62 (18, 89)	0.34
Gender (male)	59.3%	56.5%	61.1%	0.407
Type of malignancy				0.609
Hematological	61.8%	58.9%	63.7%	0.385
Solid tumor	38.2%	41.1%	36.3%	
Metastatic disease	72.7%	92.7%	74.2%	*0.035*
Active disease (no remission)	86.8%	82.3%	89.6%	0.059
Stem cell transplant (yes)	31.6%	21.8%	18.1%	0.214
Co-morbidity index ≥ 5	68.5%	66.1%	69.9%	0.475
Chemotherapy within 10 days	24.3%	28.2%	21.8%	0.190
CNS malignancy involvement*	26.5%	33.9%	21.8%	*0.017*
Medical history of seizure disorder	10.4%	16.1%	10.9%	*0.008*
Medical service	89.3%	83.0%	94.8%	*0.006*
Variables during ICU stay				
SOFA on admission**	8.5 ± 3.7, 8 (0–19)	7.2 ± 3.3, 7 (0–16)	9.3 ± 3.8, 9 (2–19)	*< 0.0001*
SOFA at time of EEG ^#^	9.9 ± 4.0, 10 (3–22)	8.2 ± 3.7, 7 (3–21)	11.1 ± 3.7, 11 (3–22)	*< 0.0001*
GCS at time of EEG^##^ (median)	7 (3–12)	8 (3–12)	7 (3–12)	*0.001*
Vasopressors (yes)	56.5%	35.5%	69.9%	*< 0.0001*
Acute renal failure (yes)	52.4%	31.5%	65.8%	*< 0.0001*
Mechanical Ventilation (yes)	79.5%	66.9%	87.6%	*< 0.0001*
Use of sedatives (yes)	49.8%	44.4%	53.4%	0.117
Cardiac arrest during ICU stay	13.3%	3.2%	19.7%	*< 0.0001*

*Central nervous system

**Sequential Organ Failure Assessment

^#^Electroencephalogram

^##^Glasgow Coma Score

**Table 4:** Multiple regression model of risk factors for mortality

Variables	Odds ratio	95% CI for OR	*p* value
Age	0.986	0.966–1.007	0.1909
Vasopressors (yes vs no)	2.245	1.188–4.245	*0.0128*
Acute renal failure (yes vs no)	2.729	1.433–5.198	*0.0023*
Improvement of GCS prior to discharge (yes vs no)	0.064	0.034–0.119	*< 0.0001*
Seizures (no vs yes)	1.595	0.687–3.703	0.2768
Medical history seizure disorder (no vs yes)	2.199	0.807–5.992	0.1234

## Discussion

Altered mental status due to non-convulsive seizures occurs in 18 to 45% of ICU patients [1–4, 13, 14]. In our study, critically ill cancer patients with moderate to severe encephalopathy have a similar incidence of NCS (14.5%) to non-cancer critically ill patients. On the contrary, the prevalence of NCSE (65%) in our study is higher than the 5% reported in medical ICUs [2] but similar to that observed in specialized neuro-ICUs [1]. In cancer patients who are not critically ill, AMS is caused by NCS in 6–9% of cases [20, 21]. In a study performed at Memorial Sloan-Kettering Cancer Center, 11.5% of lethargic and comatose patients had NCSE [22]. Differences in our patient cohort could explain the differences with other published data. More than 60% of the patients who underwent EEG monitoring in our study had multi-organ failure and SOFA scores ≥ 10. This degree of organ failure is generally associated with significant cytokine release and secondary CNS dysfunction and blood-brain barrier disruption [3, 23, 24]. Injury to the blood-brain barrier is a contributing factor for seizures [25], and this effect can be amplified in our critically ill patients as 86.7% of patients already have risk factors for seizures. Therefore, routine EEG monitoring of critically ill cancer patients with moderate to severe encephalopathy should be considered, as there is a significant prevalence of NCS and NCSE in this population.

Known risk factors for non-convulsive seizures in critically ill patients include sepsis, CNS infection, stroke, recovery from convulsive status epilepticus, and cardiac arrest [1, 5, 13, 14, 17, 20, 26]. In oncological patients, CNS involvement from malignancy, brain radiation, paraneoplastic syndromes, PRES, and medications such as chemotherapy agents and immunosuppressants is known to cause seizures [12, 13, 27]. Unlike other studies, we observed that variables such as sepsis, malignancy in the CNS, PRES, antibiotic use, anoxic brain injury, meningoencephalitis, chemotherapeutic agents, and other immunosuppressants were not associated with a higher incidence of NCS. Abnormal findings on brain imaging, however, were associated to NCS in our study, supporting already published data [5]. Remarkably, we observed that encephalopathic patients without organ failure, and specifically those with normal renal function, are more likely to have NCS as a cause for their AMS. Clinically, these findings are of great importance as our data suggests that common risk factors for seizures do not seem to have an impact in oncological patients. If an otherwise improving critically ill patient has significant encephalopathy and normal renal function, one needs to consider NCS. In these cases, quick diagnosis with EEG and AED treatment should be a priority.

Initial analysis of our patient cohort showed that administration of chemotherapy within 10 days of EEG monitoring was associated to a higher incidence of NCS. Chemotherapy has been associated to worsening mental status, and specific agents are known to cause seizures [13, 21]. Our initial analysis included patients receiving CAR T cell therapy, which is known to cause encephalopathy and NCS within 7 days of cell infusion [28, 29]. Moreover, CAR T cell patients always receive chemotherapy prior to cell infusion. With this in mind, we questioned whether the relationship between recent chemotherapy administration and NCS was due to the inclusion of CAR T cell patients in our cohort population. When controlling for CAR T cell therapy, chemotherapy was no longer an important factor in the incidence of NCS. Therefore, we can conclude that it is CAR T cell therapy, and not chemotherapy, that is the significant causative factor of seizures.

In our study, only 44% of all encephalopathic patients who underwent EEG monitoring had neurologic recovery before hospital discharge. These findings are lower than the neurological improvement observed in studies of encephalopathic patients in the ICU [1, 5, 14, 21, 22]. Moreover, when we analyzed the patients who had NCS in our study, 52% had improvement of their GCS prior to discharge. These findings are surprising as our rate of response to AEDs was more than 87%, similar to the response rate reported in the literature of both cancer and non-cancer patients [20, 22, 30]. Our cohort of patients could explain the low prevalence of improvement of GCS in this study. Encephalopathy has a negative impact in overall neurological recovery of cancer patients [21, 31]. Moreover, patients with NCS and status epilepticus are also known to have poor neurological recovery [5–8]. Therefore, our cohort population of cancer patients with ongoing moderate to severe encephalopathy, and with a significant prevalence of NCS and NCSE, could reflect on our findings of poor neurological recovery.

Besides poor neurological recovery, patients with cancer who have an underlying encephalopathy also have high morbidity and mortality [21, 31]. In our study, all encephalopathic patients who underwent EEG monitoring had prolonged ICU length of stay and high mortality. When compared to prior published data of MD Anderson’s ICU cancer patients, encephalopathic patients who underwent EEG monitoring in our study have almost three times longer ICU length of stay and higher mortality [32]. These findings indicate that encephalopathic critically ill oncological patients carry a higher morbidity and mortality when compared to all other critically ill cancer patients. Nevertheless, the findings of increased mortality cannot be explained by the presence of NCS in our studied population. Contrary to that described in the literature, mortality in patients with NCS was lower when compared to patients without seizures [3, 5–7]. First, these findings can be explained by the lower incidence of renal failure, vasopressor use, and lower SOFA scores in patients with NCS, all which are associated with increased mortality in the ICU [33, 34]. Moreover, one could suggest that an encephalopathy caused by NCS, reflects a treatable and likely reversible pathology. On the contrary, if the encephalopathy is not caused by seizures, the altered mentation is a sign of brain dysfunction associated to multi-organ failure. Therefore, in encephalopathic ICU cancer patients, NCS may be present in otherwise recovering patients and reflect a better prognosis if treated promptly. The diagnosis of NCS in critically ill cancer patients can positively impact outcomes and should not be left unrecognized.

There are some limitations to our study; the majority of EEGs performed were 20–40 min long, which could have led to under reporting of the incidence of seizures in our patient population. While data suggest that a 30-min EEG can diagnose up to 92% of patients with NCS, there is literature to support that seizures can be present after 48 h of EEG monitoring [5, 26, 35]. Second, our study focused only on patients with moderate to severe encephalopathy and those in whom the managing team decided to perform EEG monitoring. Such a cohort could lead to a bias towards worse outcomes and possibly underdiagnoses of NCS in critically ill cancer patients. Lastly, more than 49.8% of patients in the study required sedation during their ICU stay, which could have an impact in the cohort of our patient population. We observed that more patients in the non-seizure group used sedation, which should bring into consideration that sedatives, such as benzodiazepines, could have treated underlying seizures leading to bias and lower incidence of seizures. Despite this, we believe our study criteria can help intensivists decide which critically ill encephalopathic cancer patients would benefit from EEG monitoring.

## Conclusions

Encephalopathic critically ill oncological patients carry a high morbidity and mortality when compared to other patients admitted to the ICU. The incidence of NCS in this patient population is 14.5% and their presence is associated with brain imaging abnormalities and lower degree of organ failure. This cohort of patients are more likely to have lower SOFA scores and less likely to have renal failure in contrast to common clinical dictum. NCS in critically ill cancer patients with encephalopathy should lead to quick diagnosis and treatment, as these patients respond to AEDs and do not necessarily portend a worse prognosis. In contrast to other populations, non-convulsive seizures should not be used as a poor prognostic factor in critically ill encephalopathic cancer patients as the AMS reflects a reversible underlying cause, rather than a marker of irreversible multi-organ failure.

## Data Availability

Due to institutional IRB concerns, data is not publicly available; however, data may be available from the authors upon reasonable request and with permission of MD Anderson Cancer Center.

## Abbreviations

AEDs: Anti-epileptic drugs
AMS: Altered mental status
CAR: Chimeric antigen receptor
CNS: Central nervous system
EEG: Electroencephalogram
GCS: Glasgow Coma Score
ICU: Intensive Care Unit
LOS: Length of stay
NCS: Non-convulsive seizures
NCSE: Non-convulsive status epilepticus
PRES: Posterior reversible encephalopathy syndrome
SOFA: Sequential Organ Failure Assessment

## References

[1] Claassen J, Mayer SA, Kowalski RG, Emerson RG, Hirsch LJ (2004). Detection of electrographic seizures with continuous EEG monitoring in critically ill patients. Neurology..

[2] Kurtz P, Gaspard N, Wahl AS, Bauer RM, Hirsch LJ, Wunsch H (2014). Continuous electroencephalography in a surgical intensive care unit. Intensive care medicine..

[3] Oddo M, Carrera E, Claassen J, Mayer SA, Hirsch LJ (2009). Continuous electroencephalography in the medical intensive care unit. Critical care medicine..

[4] Schmitt SE (2017). Utility of clinical features for the diagnosis of seizures in the intensive care unit. J Clin Neurophysiol..

[5] Laccheo I, Sonmezturk H, Bhatt AB, Tomycz L, Shi Y, Ringel M (2015). Non-convulsive status epilepticus and non-convulsive seizures in neurological ICU patients. Neurocrit Care..

[6] Siraklow S, Trongtrakul K, Chittawatanarat K, Pathonsamit C, Teeratchanan T, Poopipatpab S (2016). The incidence, characteristics, and outcomes of stroke and seizure in critically ill surgical patients: a multicenter cohort study of Thai Surgical Intensive Care Units (THAI-SICU Study). J Med Assoc Thai..

[7] Hay A, Bellomo R, Pilcher D, Jackson G, Kaukonen KM, Bailey M (2016). Characteristics and outcome of patients with the ICU admission diagnosis of status epilepticus in Australia and New Zealand. J Crit Care..

[8] Guekht A, Mizinova M, Ershov A, Guz D, Kaimovsky I, Messina P (2015). In-hospital costs in patients with seizures and epilepsy after stroke. Epilepsia..

[9] Punia V, Garcia CG, Hantus S (2015). Incidence of recurrent seizures following hospital discharge in patients with LPDs (PLEDs) and nonconvulsive seizures recorded on continuous EEG in the critical care setting. Epilepsy Behav..

[10] Arif H, Hirsch LJ (2008). Treatment of status epilepticus. Seminars in neurology..

[11] Chen JW, Wasterlain CG (2006). Status epilepticus: pathophysiology and management in adults. The Lancet Neurology..

[12] Damek DM (2010). Cerebral edema, altered mental status, seizures, acute stroke, leptomeningeal metastases, and paraneoplastic syndrome. Hematology/oncology clinics of North America..

[13] Grewal J, Grewal HK, Forman AD (2008). Seizures and epilepsy in cancer: etiologies, evaluation, and management. Current oncology reports..

[14] Riedijk M, van den Bergh WM, van Vliet M, Kusadasi N, Span LR, Tuinman PR (2015). Characteristics and outcomes of patients with a haematological malignancy admitted to the intensive care unit for a neurological event. Crit Care Resusc..

[15] Harris PA, Taylor R, Thielke R, Payne J, Gonzalez N, Conde JG (2009). Research electronic data capture (REDCap)--a metadata-driven methodology and workflow process for providing translational research informatics support. J Biomed Inform..

[16] Leitinger M, Beniczky S, Rohracher A, Gardella E, Kalss G, Qerama E (2015). Salzburg consensus criteria for non-convulsive status epilepticus--approach to clinical application. Epilepsy Behav..

[17] Sutter R, Ruegg S, Tschudin-Sutter S (2015). Seizures as adverse events of antibiotic drugs: a systematic review. Neurology..

[18] Gilmore EJ, Gaspard N, Choi HA, Cohen E, Burkart KM, Chong DH (2015). Acute brain failure in severe sepsis: a prospective study in the medical intensive care unit utilizing continuous EEG monitoring. Intensive care medicine..

[19] Misra UK, Kalita J, Chandra S, Nair PP (2013). Association of antibiotics with status epilepticus. Neurol Sci..

[20] Cocito L, Audenino D, Primavera A (2001). Altered mental state and nonconvulsive status epilepticus in patients with cancer. Arch Neurol..

[21] Tuma R, DeAngelis LM (2000). Altered mental status in patients with cancer. Arch Neurol..

[22] Spindler M, Jacks LM, Chen X, Panageas K, DeAngelis LM, Avila EK (2013). Spectrum of nonconvulsive status epilepticus in patients with cancer. J Clin Neurophysiol..

[23] Ebersoldt M, Sharshar T, Annane D (2007). Sepsis-associated delirium. Intensive care medicine..

[24] Bleck Thomas P. (2018). How Critical Illness Affects the Brain…and Vice Versa. Critical Care Medicine.

[25] Marchi N, Angelov L, Masaryk T, Fazio V, Granata T, Hernandez N (2007). Seizure-promoting effect of blood-brain barrier disruption. Epilepsia..

[26] Claassen J, Taccone FS, Horn P, Holtkamp M, Stocchetti N, Oddo M (2013). Recommendations on the use of EEG monitoring in critically ill patients: consensus statement from the neurointensive care section of the ESICM. Intensive care medicine..

[27] Gutierrez C, Pastores S. Oncologic emergencies. 4th ed. JB H, GA S, JP K, editors: McGraw-Hill Medical; 2015.

[28] Gutierrez C, McEvoy C, Mead E, Stephens RS, Munshi L, Detsky ME (2018). Management of the critically ill adult chimeric antigen receptor T cell therapy patient: a critical care perspective. Critical care medicine..

[29] Neelapu SS, Tummala S, Kebriaei P, Wierda W, Gutierrez C, Locke FL (2018). Chimeric antigen receptor T-cell therapy - assessment and management of toxicities. Nat Rev Clin Oncol..

[30] Rudin D, Grize L, Schindler C, Marsch S, Ruegg S, Sutter R (2011). High prevalence of nonconvulsive and subtle status epilepticus in an ICU of a tertiary care center: a three-year observational cohort study. Epilepsy Res..

[31] Tabouret E, Boucard C, Devillier R, Barrie M, Boussen S, Autran D (2016). Neuro-oncological patients admitted in intensive-care unit: predictive factors and functional outcome. J Neurooncol..

[32] Wallace SK, Rathi NK, Waller DK, Ensor JE, Haque SA, Price KJ (2016). Two decades of ICU utilization and hospital outcomes in a comprehensive cancer center. Critical care medicine..

[33] Darmon M, Bourmaud A, Georges Q, Soares M, Jeon K, Oeyen S (2019). Changes in critically ill cancer patients' short-term outcome over the last decades: results of systematic review with meta-analysis on individual data. Intensive care medicine..

[34] Ferreira FL, Bota DP, Bross A, Melot C, Vincent JL (2001). Serial evaluation of the SOFA score to predict outcome in critically ill patients. JAMA..

[35] Khan OI, Azevedo CJ, Hartshorn AL, Montanye JT, Gonzalez JC, Natola MA (2014). A comparison of continuous video-EEG monitoring and 30-minute EEG in an ICU. Epileptic Disord..

